# How does attendance at Schwartz Rounds affect veterinary professionals' experience in clinical practice?

**DOI:** 10.1002/vetr.70217

**Published:** 2026-04-02

**Authors:** Robert Campbell, Emma Ormandy

**Affiliations:** ^1^ School of Veterinary Science University of Liverpool Liverpool UK

## Abstract

**Background:**

Schwartz Rounds are a group reflective forum for healthcare staff to openly and safely discuss the emotional aspects of their work. With the plethora of challenges facing veterinary professionals, any organisational intervention that could help support team wellbeing should be evaluated to provide evidence for any beneficial claims.

**Methods:**

Ten veterinary professionals who had attended two or more Schwartz Rounds in clinical practice were interviewed to explore their experience with the initiative. Transcripts were processed via reflexive thematic analysis to generate themes.

**Results:**

Three themes were generated: ‘enabling compassion’, ‘empowering vulnerability’ and ‘enhancing organisational insight’. Schwartz Rounds attendees showed compassion for themselves and others through shared storytelling and active listening. Witnessing senior colleagues showcased vulnerabilities, built team connectedness and helped humanise leaders. Finally, Schwartz Rounds provided attendees with organisational insight into the wider workplace experience of others.

**Limitations:**

Non‐clinical team members’ experiences were not included in this study. Impacts on patient care and client experience would broaden clinical practice insights.

**Conclusions:**

There is evidence to show that Schwartz Rounds positively impact the working conditions of veterinary professionals in clinical practice and leads to changes in workplace culture through shared understanding, improved colleague relationships and enabling compassion in the workplace.

## INTRODUCTION

Veterinary professionals in the UK include veterinary surgeons and registered veterinary nurses (RVNs), and both are governed by the RCVS.^1^ Although the roles in which veterinary professionals work in have diversified in recent years, the majority remain in clinical practice.^2^, ^3^ The provision of veterinary care can be emotionally challenging, with recently surveyed veterinary professionals stating that 90% of veterinarians and 82% of RVNs considered their work to be stressful.^4^, ^5^

Insights into veterinary workplace stressors are well documented; however, they vary between individuals.^6^ High workload is commonly recognised as an organisational workplace stressor for veterinary staff, which has only increased following a rise in pet ownership during the COVID‐19 pandemic.^7^ Beyond workload volume, there are many other contributing stressors for those working in practice. Organisational stressors such as poor work‒life balance, staff shortages, long hours and low remuneration as well as individual workplace stressors, including client interactions, colleague incivility and fear of making mistakes, all add to the daily emotional toll placed on veterinary professionals.^6^, ^8^, ^9^

Those working in clinical practice consistently survey as having below‐average mental wellbeing scores and being at increased risk of burnout.^10^, ^11^, ^12^ Since 2010, the RCVS has monitored mental wellbeing scores in the profession, and a consistent decline has been viewed over the past 15 years.^2^, ^3^, ^12^, ^13^, ^14^ This garners particular concern, especially among a workforce that is documented to have a three times greater suicide rate than the UK general population.^15^

The RCVS has taken action to address the wellbeing of the profession, with the establishment of the Mind Matters Initiative in 2015. Mental wellbeing now forms part of the RCVS Practice Standards Scheme, an initiative to formally accredit UK veterinary practices.^16^ The British Veterinary Association also offers their Good Veterinary Workplaces accreditation to highlight positive workplace culture, with specific reference made to fostering psychological safety and respecting mental and physical health equally.^17^

Dealing with staff shortages was consistently ranked in the top three workplace stressors by both veterinary surgeons and veterinary nurses.^6^ Surveys revealed that offering more flexible working opportunities, such as part‐time and term‐time contracts, as well as homeworking and condensed hours, could help with staff retention.^18^

Veterinary incivility incidences in clinical practice are often cited as individual workplace stressors, and Irwin et al. have contributed to a better understanding of this issue, leading to the publication of a veterinary incivility toolkit to be used in veterinary practices.^19^, ^20^

There is growing work investigating the possible wellbeing benefits of facilitated group reflection through post‐event clinical debriefings and virtual ethics rounds.^21^, ^22^ These look to bring the veterinary team together to critically evaluate ethical and traumatic cases and develop plans for future improvement. Studies evaluating these forums have found that they improve team members’ ability to navigate ethically challenging situations, potentially helping to mitigate moral distress among veterinary professionals.^23^

### Human healthcare comparison

While the evidence for poor mental wellbeing in the veterinary profession is well established, research evaluating the effectiveness of interventions remains limited. Workplace stressors experienced by veterinary professionals and human healthcare staff share similarities and differences; therefore, exploring workplace wellbeing initiatives that are effective in human healthcare settings can be a worthwhile pursuit for advancing the veterinary knowledge base.

The 2024 National Health Service survey found 41% of staff were unwell due to work‐related stress.^24^ Common workplace stressors experienced by human healthcare workers include workload pressure, long hours, moral conflicts and workplace‐related bullying.^25^ The potential consequences of these stressors are evidenced by an increased risk of burnout, depression and anxiety in human healthcare providers, mirroring that of what is recognised in veterinary professionals.^25^, ^26^, ^27^

Making comparisons between the two care‐giving workforces is helpful to establish a research rationale but careful consideration needs to be afforded for key differences between the professions. Major differences, such as payment models, service provisions, practitioner–patient relationships and workforce training, mean that workplace wellbeing initiatives developed for human healthcare cannot be assumed to work in the veterinary profession without robust evaluation.

This study therefore aims to build upon the evidence base for a workplace wellbeing initiative first intended for human healthcare providers but now being used in the veterinary profession. Building on the quantitative research published by Martin,^28^ this study aims to provide a qualitative insight into the experience of veterinary professionals attending Schwartz Rounds while working in clinical practice.

### Schwartz Rounds

Schwartz Rounds (or simply ‘Rounds’) are a group reflective forum in which staff and students come together in a safe and confidential environment to discuss the emotional and social aspects of their work in health and care.^29^ Originally conceived in Boston, USA, Schwartz Rounds are the legacy of Kenneth Schwartz, a healthcare lawyer who was diagnosed with terminal cancer. During his treatment, he wrote of the compassionate care he received in his final days and how ‘the smallest acts of kindness made the unbearable bearable’.^30^

Since their introduction to the UK in 2009, Schwartz Rounds have expanded to approximately 180 organisations throughout the UK and Ireland. Rounds have a strong presence across the NHS, but also run in hospice care, social care and higher education institutions.

Schwartz Rounds are open groups available to all staff in an organisation. Rounds last for one hour and start with a panel of storytellers retelling a personal experience from their work related to a chosen theme; for example, ‘A patient I'll never forget’. Following this, the audience is then invited to contribute with personal resonance, shared storytelling and empathetic listening. Audience discussion is carefully facilitated by two trained Schwartz Rounds facilitators, who ensure that emotional containment and psychological safety are maintained throughout.^31^, ^32^

Schwartz Rounds can only be run under a license. The license holder for the UK and Ireland has been the Point of Care Foundation (PoCF), a not‐for‐profit UK charity. However, from 1 January 2026, the Schwartz Center for Compassionate Healthcare in Boston, USA, will provide the license, training and support for Schwartz Rounds in the UK and Ireland.

Noting the positive benefits enjoyed by attendees of Schwartz Rounds in the NHS, the RCVS Mind Matters Initiative funded a pilot study of veterinary centres adopting Rounds in 2019.^33^ One such centre was the University of Liverpool School of Veterinary Science. Through a qualitative investigation, this study aimed to gain further understanding of the impact of attending Schwartz Rounds on veterinary nurses and veterinary surgeons working in clinical practice. The research questions proposed were as follows:

1. In what ways does attendance at Schwartz Rounds impact the experience of veterinary professionals working in clinical practice?
2. Are the proven benefits of Schwartz Rounds in human healthcare translatable to those working in the veterinary profession?

## MATERIALS AND METHODS

### Data collection

An open advertising approach was used to reach potential research participants. An advertisement poster was disseminated online by the PoCF, as well as the universities’ alumni services to reach the eligible study population; veterinary professionals (veterinary nurses and veterinary surgeons) who had attended two or more Schwartz Rounds while working in clinical practice.

Interested participants were invited to contact the lead researcher (R.C.), and then a participant information sheet was sent alongside a consent form (Supporting Information). Individual interviews were used due to the emotional and personal nature of conversations during Rounds, which may not be as forthcoming in a focus group setting.^34^ The interviews were conducted by R.C. and carried out online and automatically transcribed using Microsoft Teams built‐in transcription software (version 25060.203.3471.5023).

The interviews followed a semi‐structured schedule, allowing flexibility in discussion while still following a methodical order of inquiry. The question prompts were pilot tested with a qualitative researcher prior to interviews commencing. The participants had seven days to withdraw their involvement following interview. The transcripts were then anonymised and securely stored as per the University of Liverpool's Research Data Management policy.^35^

### Data analysis

Qualitative research methods were utilised to help capture rich insights from those attending Schwartz Rounds.^36^

The interview transcripts were analysed following Braun and Clarke's reflexive thematic analysis (RTA) methodological approach, moving through six phases of a recursive and flexible qualitative process.^37^, ^38^ The interview transcripts were re‐read multiple times to support familiarisation with the data.

Following familiarisation, initial coding took place. Codes were defined as any data item that may contribute to answering the research questions.^39^ As more interviews commenced, further codes were generated—both semantic and latent in nature. Codes were then organised around a central, core commonality, generating themes as fully realised stories of meaning. The final themes were then defined and named. Table 1 details an exemplar coding process from transcript data to theme generation.

**TABLE 1:** Reflective thematic analysis coding process.

Transcript extract	Code	Sub‐theme	Theme
‘I don't think I expected the feeling to be so profound, actually. The feeling of processing and letting go of something that I've been carrying and as we know most of these stories, people have been carrying for years and years and years and yeah, it felt really profound’	The process of being a storyteller during a Schwartz Rounds is cathartic and the act of proclaiming the story is emotionally purging and healing.	Impact on the veterinary professional	Enabling compassion

### Reflexive statement

Researcher reflexivity is a crucial part to performing good qualitative inquiry. An RTA methodology enlists the participant and researcher as co‐collaborators for the research. It is vital that researchers take measures to be self‐aware of their inherent biases and assumptions while advancing through the RTA process.^40^ The lead researcher documented their reflexivity throughout the data collection process and logged journal entries following each interview.

### Ethical acknowledgement

The emotional nature of conversations in Rounds was expected to emerge during interviews. Careful consideration was made to maintain psychological safety throughout, with participants being reminded of their right to withdraw from the study at any time prior to anonymisation. The participants were additionally signposted to mental health support post‐interview.

## RESULTS

### Interview process

Ten individual semi‐structured interviews were performed with six veterinary surgeons and four veterinary nurses between 26 February and 21 May 2024 and lasted 25‒50 minutes. Data saturation was reached prior to the 10th interview; hence, no further interviews were performed. The participants practiced in a range of clinical settings and had varied lengths of clinical experience, as displayed in Table 2.

**TABLE 2:** Participant demographics.

Participant no.	Gender	Job role	Area of practice	Years in practice
# 1	F	Veterinarian	Small animal	5‒10
# 2	F	Veterinary nurse	Small animal	5‒10
# 3	F	Veterinarian	Equine	10‒15
# 4	F	Veterinarian	Small animal	20+
# 5	F	Veterinary nurse	Small animal	15‒20
# 6	F	Veterinarian	Small animal	10‒15
# 7	F	Veterinary nurse	Small animal	20+
# 8	F	Veterinary nurse	Small animal	20+
# 9	F	Veterinarian	Equine	20+
# 10	M	Veterinarian	Equine	20+

### Themes

Following transcript analysis, three themes were generated and further defined to connect individual interview findings and answer the research questions posed.

1. Enabling compassion—the ability to activate compassion between others and with oneself.
2. Empowering vulnerability—encouraging a space where attendees could own their anxieties and failings.
3. Enhancing organisational insight—functioning as a practice‐wide emotional barometer for attendees and non‐attendees.

### Theme one: Enabling compassion

Compassion, ‘the awareness of other's suffering and a desire to relieve it’,^41^ was multidimensional among attendees and directed towards fellow colleagues, pet carers and oneself.

Participant 8 noted how expressing emotions during Rounds led to greater understanding from colleagues of the impact that workplace stressors had on them:

… no one's going to look at the fact that you're running behind and go, ‘you're running behind again!’ because we all know that it happens and we all know to help each other out. And it's lovely because we've all expressed how frustrated and overwhelmed we get when that happens, when we're in Schwartz Rounds. (Participant 8)

Another example of colleague‐directed compassion following Rounds was an openness to supporting colleagues experiencing psychological distress.

I think it's made me more comfortable around emotion and around discomfort, and so it's easier to sit with someone who's distressed and not intervene because that's not always what people want. (Participant 7)

Participant 7 felt that some of the key components of Schwartz Rounds helped them be a listening friend towards colleagues. Further excerpts from interviews showed that compassion was extended towards clients too. Often in clinical practice, there is an expectation to emotionally detach from clients:

And actually what Schwartz Round teaches us is that, the moments of compassion where we crossover that barrier are the things that are the most important to our ‘patients’ in human healthcare and ‘clients’ in the veterinary world. (Participant 7)

Through attending Rounds, participant 7 felt that there was a greater appreciation for displaying humanity towards clients rather than maintaining a strictly professional front.

Beyond colleagues and clients, self‐compassion was also documented among attendees. Participant 3 spoke of how hearing others’ experiences helped validate their own emotions, and led them to enact self‐compassion when seeking professional help for feelings of imposter syndrome:

I developed a deep sense of imposter syndrome … I did think back to that very first Schwartz Round and how I remember a lot of people admitting that and I think having had that experience in Schwartz Rounds, that empowered me to get some help for that. So I did contact Vetlife and I talked to them about it …. (Participant 3)

Participant 8 also spoke of the power of resonating with others’ emotions during Rounds and how this helped authenticate her own emotions while quashing thoughts of self‐doubt.

And yeah, I think just the talking about it in itself was very purging, … but it was the team support that did the healing and made me realise that I wasn't just being a numpty. (Participant 8)

### Theme two: Empowering vulnerability

The second theme generated was the feeling of encouragement and safety among attendees in openly discussing their failings, anxieties and shortcomings in the workplace.

Those in leadership roles spoke of how Schwartz Rounds and the therapeutic nature of discussions, allowed more experienced team members to feel supported when showcasing their vulnerabilities to colleagues. Participant 7 references back to a time when they were anxious prior to working a nursing shift after having had time away from clinical work.

I think that [having Schwartz Rounds] benefited the staff because it reminded them that we don't all know everything and you know it's a good way for a leader to be vulnerable. It's very difficult to practise when you're used to kind of trying to know everything and be perfect. The Schwartz Rounds and the way that we connect together as a team enabled me to go in like that in a more vulnerable state. (Participant 7)

Similar feelings of relief and acceptance upon displaying vulnerabilities were felt by participant 9, who is a senior clinician with over 20 years of clinical experience.

I think it's just that opening you up you know and that sort of mask that you have as a veterinary professional sort of being shifted a little bit. That feeling of slight vulnerability and feeling slightly uncomfortable when publicly discussing your emotions. I think just that feeling of vulnerability, but also a little bit of a sense of relief. Just sort of telling that story. (Participant 9)

Participant 4 was also a senior veterinarian and saw Schwartz Rounds as an opportunity for leaders to open up to less experienced colleagues in a way that humanised them:

Also, we don't always know the answers. I think sometimes realising that we're all fallible and we make mistakes. Sometimes I do think some of the younger ones are like ‘oh, but you must know everything’, and you're like, we don't, we really don't. We're making it up sometimes. You know, a lot of the time. (Participant 4)

Attendees who held more junior positions in the workplace were reminded during Schwartz Rounds that their seniors were human too. Participant 8 spoke of her surprise when seeing a highly regarded senior colleague exposing vulnerability and how this made her admire them even more.

One of the vets who I always thought was hard as nails was crying and that blew my mind because she was the last person that I ever thought of crying or feeling that sort of emotion about anything. She was fabulous. (Participant 8)

Participant 2 shares this sentiment, and mentions how seeing leaders showcase emotions can help to build feelings of connectedness among assumed hierarchy.

I think you look up to some of these people in the profession and don't potentially see them as real people, until you see them in a situation where you can relate to them and understand that actually no, they've been in situations that you've been in and they've felt the emotions that you felt. They are normal people, they are humans. (Participant 2)

Rounds helping to humanise role models was seen to be a profoundly progressive step for onlookers who felt their feelings of vulnerability had been validated:

He [a colleague] was someone that I really, really looked up to and always thought he had the most fantastic coping mechanisms. So to hear him having gone through something fairly similar as well, or something that was upsetting for him, humanised him a little bit more and therefore made me feel slightly less silly for being emotional about something. (Participant 8)

Displays of vulnerability from the senior management team led to shifts in workplace dynamics inside and outside the Rounds environment. Participant 1 felt that when seeing their manager openly discuss emotions during Schwartz Rounds, this opened the door for team members to request meetings to discuss struggles they may be experiencing.

I think from a management point of view like in my practice manager's perspective, she's kind of opening the floor to everyone, giving people the opportunity to feel probably a little bit more comfortable that if they were mentally struggling that they could book a one to one with her to express that they were. (Participant 1)

### Theme three: Enhancing organisational insight

The final theme constructed was the impact that attending Schwartz Rounds had on understanding the wider veterinary team and the organisation in which they work. Attendance of Rounds is voluntary, and this was seen to divide the workplace into attendees and non‐attendees. This participant expands:

I think it looks like the practice almost sort of divides in two. There are some people that always go. And there are some that just keep away and don't want to get involved because it's either too fluffy or too awkward, or actually sometimes I think probably there are people who could really benefit from going but don't want to open up. (Participant 4)

Participant 4 understands if engaging with Rounds is too emotionally challenging for non‐attendees but feels there is still a benefit to be gained among the struggle. For attendees, Rounds offered an opportunity for team members to learn about one another through meaningful conversation that otherwise may not have occurred:

It's a really big team with multiple practices and you don't get to see or meet everyone. So I remember it being good to get to know individuals that maybe I haven't really met or spoken to a lot. So for me that was quite helpful coming into a really big practice and getting to know everybody a little bit more at a slightly more personal level. (Participant 6)

Participant 6 attended Rounds when she was new to her team, allowing an opportunity to gain more insight into her colleagues’ backgrounds. Participant 8 also felt more connected to her wider team following Rounds, especially those working in satellite clinics:

It's really built a bond between me and a couple of the staff that I would never have otherwise even spoken to because they're always in different branches. And then it turns out that they're the most magnificent people and they've got this beautiful soul that I just never knew about. So yeah, there's a lot of community spirit I think that comes out of it and realising that we are really all in the same boat. (Participant 8)

The conversations taking place during Rounds were noted as being much deeper in nature than would usually be had among the veterinary team:

You know, [in my previous practice with no Schwartz Rounds] we rarely talked about the emotional side of things, unless it was anger. So you know, we're frustrated with a client. That was a common theme in our conversations, but not really the harder aspects like you talk about in Schwartz Rounds. (Participant 3)

Participant 3 compares how when working in a practice without Schwartz Rounds, conversations between colleagues were only ever superficial in nature. Participant 9 referred to how strict adherence to the Schwartz Rounds model helps foster psychological safety:

[when displaying] that feeling of slight vulnerability and feeling slightly uncomfortable sort of publicly discussing your emotions. And that's where Schwartz Rounds are great in that it is put into a framework where it does feel very safe and structured, which I think otherwise it could turn into a negative thing. (Participant 9)

Through these conversations, it was noted that Rounds allow colleagues to gain insights into other job roles among the organisation, especially non‐clinical roles:

Going to Schwartz Rounds and listening to the people, I've really got more of an idea of how some of the lay staff in particular feel about their job or what they do and don't know … I remember one there was quite an experienced receptionist, but she was saying when she came in she had had no idea what we did with bodies after euthanasias. (Participant 4)

Participant 4 went on to discuss how following this interaction during Schwartz Rounds, the practice implemented mandatory euthanasia training for all new receptionists. This example of positive change following improved colleague understanding was then noted again when members of the veterinary team shared personal insights during Rounds that impacted their day‐to‐day work:

we're just finding out fascinating things about people that have talked about religious stuff, which I would never have thought of or thought they were, you know, thinking about neurodiverse stuff. I'm just like, oh, God, I've not clocked that at all. And then it's made me think, oh, actually. We do need to consider that. So, it definitely has longer term effects afterwards. (Participant 4)

Participant 4 was a member of their practice's senior management team and was particularly interested in insights from minority‐group team members that could improve the organisation's working culture.

## DISCUSSION

The findings gathered from the participant interviews provide the first qualitative insight into veterinary professionals attending Schwartz Rounds in clinical practice in the UK. All findings share similarities with those found in human healthcare research.

Veterinary professionals felt that following attendance at Schwartz Rounds, they were able to show compassion towards others in their workplace. Transcripts reference how key aspects of the Schwartz Round model, such as shared storytelling, active listening and embracing of silences enabled compassionate interactions to take place outside of Rounds, known as a ‘ripple effect’.^29^, ^42^ This sentiment is echoed in human healthcare research where increased empathy and compassion for colleagues and patients were reported by those attending Rounds.^14^

Enabling self‐compassion was also a key component of the Rounds experience for veterinary professionals. The three components of self‐compassion are considered to be kindness, mindfulness and common humanity.^43^ All three were nurtured during Schwartz Rounds, as veterinary professionals described their experience as ‘emotionally purging’, ‘cathartic’ and ‘calming’. Human healthcare literature recognises simply making the time to attend Rounds as an act of self‐compassion amidst busy clinical responsibilities.^44^ Having this outlet to practice self‐compassion, at least monthly within the workplace, can help to reduce the build‐up of compassion stress among attendees and reduce the likelihood of emotional fatigue occurring over a lifelong veterinary career.^45^

During Rounds, the psychological safety and emotional containment of the forum's model were recognised as pre‐requisites for empowering vulnerability among attendees. Expressions of vulnerability by leaders and role models helped humanise them to other team members, building community within the workplace. This levelling of hierarchy has been documented in human healthcare settings running Rounds as well as universities.^46^, ^47^, ^48^ There is evidence to suggest that inclusion of clinical leadership is essential to the health and sustainability of good Schwartz Rounds, with organisational buy‐in fundamental to their longevity.^29^, ^49^

Attendees in this study felt that when their senior management team attended and exposed vulnerabilities during Schwartz Rounds, this was seen to reassure and welcome those requiring more formal workplace assistance to reach out privately. This is a positive sign that although Rounds are not a panacea for mental health, when properly implemented and championed, they are capable of better connecting avenues of support between professional help and colleagues in need.^50^

Schwartz Rounds were highlighted as being divisive by some participants, stating that attendee and non‐attendee cohorts existed. This is well documented in human healthcare literature, and compounding factors include Rounds being voluntary, timing issues and their proposed impact being cumulative.^29^ A self‐perpetuating cycle exists whereby those who do not attend, or attend only once, may not perceive any benefit and choose not to return. Regardless of the reason for non‐attendance, it must be assumed that there will always be team members who do not engage; therefore, an organisation must utilise alternative initiatives. These can sit side by side, complementing Schwartz Rounds in cultivating a practice‐wide culture that prioritises the wellbeing of the team as they provide veterinary care.

Further organisational insights were uncovered during Schwarts Rounds for those who did attend. For large veterinary practices, which were made up of multiple branches, Rounds offered an opportunity for team members to engage in meaningful conversation that would otherwise not take place. The coming together of clinical and non‐clinical attendees also allowed for better understanding of individual job roles that may not have connected emotionally otherwise.^44^

With improved clarity between roles, this offered attendees insights into individual working differences and interdepartmental challenges—leading ultimately to real change in onboarding processes, staff training and acknowledging a diverse practice culture. The human healthcare literature also recognises the benefit Rounds provide to better understanding non‐clinical roles, and welcoming their perspectives in workplace discussions, which are often dominated by clinical matters.^49^, ^51^

### Limitations

Schwartz Rounds are inclusive in their nature, and as such, the research produced from them should look to gather experiences from across the entire veterinary team. Future research should look to include those in non‐clinical roles and training positions.

This study focused on those who chose to attend Rounds and thus lacks insights from non‐attendees. Human healthcare studies have looked to better understand barriers to attendance, but it would be worthwhile investigating if these exist in veterinary too.

Although the lengths of research participants’ clinical careers were varied, there was an absence of those with less than five years of clinical experience. This group has been identified as more likely to leave the profession due to stress and lack of reward; therefore, without their experiences to gather evidence from, it is difficult to determine if the benefits of attending Schwartz Rounds would pertain to them too.

The cost of implementing and maintaining the initiative was not focused on during this study, but is of course a key factor in veterinary organisations adopting Schwartz Rounds.

## CONCLUSION

The first qualitative insights into the experience of veterinary professionals attending Schwartz Rounds show a similar benefit from studies involving human healthcare attendees. Veterinary surgeons and veterinary nurses feel that Schwartz Rounds have positive impacts on the veterinary professionals attending, the wider veterinary team and the organisation in which they are supported. Documenting these qualitative findings, alongside pre‐existing quantitative data, is hoped to form a foundation upon which future research can be conducted, thereby allowing informed, evidence‐based decisions to be made when considering the implementation of veterinary Schwartz Rounds.

## AUTHOR CONTRIBUTIONS

Robert Campbell and Emma Ormandy designed the study together. Robert Campbell oversaw the participant recruitment, conducted the participant interviews and performed the thematic analysis of the transcripts. Emma Ormandy reviewed the thematic analysis, and both authors collaborated to generate the final themes.

## CONFLICT OF INTEREST STATEMENT

The authors declare they have no conflicts of interest.

## FUNDING INFORMATION

The authors received no specific funding for this work.

## ETHICS STATEMENT

Following ethical approval by the University of Liverpool's Institute of Infection, Veterinary and Ecological Sciences Research Ethics Committee on 14 February 2024 (ref. 13337), data collection commenced on 26 February 2024.

## Data Availability

The data that support the findings of this study are available on request from the corresponding author. The data are not publicly available due to privacy or ethical restrictions.
